# Implementation strategies for decentralized management of multidrug-resistant tuberculosis: insights from community health systems in Zambia

**DOI:** 10.1186/s13690-024-01384-4

**Published:** 2024-09-14

**Authors:** Joseph Mumba Zulu, Patricia Maritim, Hikabasa Halwiindi, Malizgani Paul Chavula, Margarate Munakampe, Tulani Francis L. Matenga, Chris Mweemba, Ntazana N. Sinyangwe, Batuli Habib, Mwiche Musukuma, Adam Silumbwe, Bo Wang, Patrick Kaonga, Mwimba Chewe, Ronald Fisa, Jeremiah Banda, Angel Mubanga, Henry Phiri

**Affiliations:** 1 Department of Health Promotion and Education, School of Public Health, University of Zambia, PO Box 50110, Lusaka, Zambia; 2 Department of Health Policy and Management, School of Public Health, University of Zambia, PO Box 50110, Lusaka, Zambia; 3 Department of Community and Family Medicine, School of Public Health, University of Zambia, PO Box 50110, Lusaka, Zambia; 4 Department of Epidemiology and Global Health, Umeå University, Umeå, 901 87 Sweden; 5 Yakini Health Research Institute, Lusaka, Zambia; 6 Department of Environmental Health, School of Public Health, University of Zambia, PO Box 50110, Lusaka, Zambia; 7 Department of Epidemiology and Biostatics, School of Public Health, University of Zambia, PO Box 50110, Lusaka, Zambia; 8 Department of Population and Quantitative Health Sciences, University of Massachusetts Chan Medical School, 55 N Lake Ave, 01655 Worcester, MA USA; 9 Ministry of Health, Lusaka, Zambia

**Keywords:** Implementation strategies, Community health systems, Decentralized programmatic management of multidrug-resistant tuberculosis, Zambia

## Abstract

**Background:**

Decentralized management approaches for multi-drug-resistant tuberculosis (MDR TB) have shown improved treatment outcomes in patients. However, challenges remain in the delivery of decentralized MDR TB services. Further, implementation strategies for effectively delivering the services in community health systems (CHSs) in low-resource settings have not been fully described, as most strategies are known and effective in high-income settings. Our research aimed to delineate the specific implementation strategies employed in managing MDR TB in Zambia.

**Methods:**

Our qualitative case study involved 112 in-depth interviews with a diverse group of participants, including healthcare workers, community health workers, patients, caregivers, and health managers in nine districts. We categorized implementation strategies using the Expert Recommendations for Implementing Change (ERIC) compilation and later grouped them into three CHS lenses: programmatic, relational, and collective action.

**Results:**

The programmatic lens comprised four implementation strategies: (1) changing infrastructure through refurbishing and expanding health facilities to accommodate management of MDR TB, (2) adapting and tailoring clinical and diagnostic services to the context through implementing tailored strategies, (3) training and educating health providers through ongoing training, and (4) using evaluative and iterative strategies to review program performance, which involved development and organization of quality monitoring systems, as well as audits. Relational lens strategies were (1) providing interactive assistance through offering local technical assistance in clinical expert committees and (2) providing support to clinicians through developing health worker and community health worker outreach teams. Finally, the main collective action lens strategy was engaging consumers; the discrete strategies were increasing demand using community networks and events and involving patients and family members.

**Conclusion:**

This study builds on the ERIC implementation strategies by stressing the need to fully consider interrelations or embeddedness of CHS strategies during implementation processes. For example, to work effectively, the programmatic lens strategies need to be supported by strategies that promote meaningful community engagement (the relational lens) and should be attuned to strategies that promote community mobilization (collective action lens).

**Supplementary Information:**

The online version contains supplementary material available at 10.1186/s13690-024-01384-4.

**Table:** 

**Text box 1. Contributions to the literature**
• Although decentralization of management of MDR TB has shown improved treatment outcomes, challenges remain in delivery of decentralized MDR TB services, particularly in community health systems.
• Implementation strategies required to effectively deliver MDR TB services in community health systems in low-resource settings are rarely documented, as most known and effective strategies only apply to high-income settings.
• We build on the ERIC implementation strategies by stressing the need to fully consider interrelations or embeddedness of community health systems when developing and implementing strategies for decentralized MDR TB services.
• The mapping of community health system implementation strategies could enhance delivery of MDR TB services by helping experts comprehend and navigate the complex double embedded nature of these strategies in both health facility and community settings.

## Introduction

Tuberculosis (TB) remains one of the leading infectious causes of death globally despite concerted efforts to prevent transmission and provide treatment [1]. According to the 2023 Global Tuberculosis Report, 7.5 million new TB diagnoses, 10.6 million people who developed TB, and 1.30 million TB-associated deaths were reported in 2022 [1]. About 85% of people who develop TB can be successfully treated with a 6-month drug regimen of rifampicin and isoniazid or through regimens of 1–6 months if they develop multidrug-resistant or rifampicin-resistant TB (MDR/RR TB) [3]. Resistance to treatment is a growing public health concern: 410,000 people developed MDR TB in 2022 [1], comprising 3.3% of new cases and 17% of patients who had received treatment previously [2].

Zambia ranks 21st among the 30 priority countries [2], with a high burden of TB, HIV-associated TB, and MDR TB [5]. As of 2019, its estimated burden of TB was 59,000 cases [3, 4].

In WHO End TB strategy for providing integrated patient-centered care and prevention, a key pillar entails treating all people with TB, including those with drug-resistant TB. However, of people diagnosed with MDR TB in 2022, only 42.8% started treatment [1]. One approach for extending the reach of MDR TB services to underserved populations, to enhance diagnosis and treatment, involves decentralized service delivery [1]. This approach delivers a comprehensive package of services at primary-care-level rather than at national- and provincial-level treatment centres [1]. It involves caring for patients closer to their homes, in district hospitals as opposed to distant specialist regional TB centers [5]. Engagement of communities, civil society organizations, and public and private care providers is another key pillar of the decentralized approach and End TB strategy [6].

In several studies, decentralization has resulted in higher treatment success [6–10]. Specifically, it has increased patient enrollment, reduced delays in initiating treatment, and improved follow-up in communities, as well as reducing treatment failure and mortality [6–10]. For example, in a recent national assessment of the impact of decentralized programmatic management of multidrug-resistant tuberculosis in Zambia, half (50%) of the facilities recorded better patient outcomes, including more patients (547) who received services after the decentralization, compared with 148 before decentralization [7]. In some countries, decentralization significantly reduced treatment costs [8].

Zambia began treating drug-resistant TB patients following certification from the green light committee (GLC) in 2009 [2]. This was primarily done by setting up two drug-resistant TB treatment centers, the University Teaching Hospital (UTH) in Lusaka District and Ndola Teaching Hospital in Ndola District. Until 2018, these two centres admitted all drug-resistant TB patients for the entire 8 months of the intensive phase, posing challenges to patients who had limited access to the facilities. The substantial number of referrals delayed initiation of treatment and heightened demand on the ambulance services required for referral processes [9].

Like other complex health interventions, decentralized management synergizes resources from community-based and formal health systems. It frequently operates in a grey zone, “intersecting and navigating the domains of public, non-governmental, and private health sectors*”* [10]. Community health systems (CHS) can play a crucial role in delivery and acceptability, as decentralizations aim to achieve universal health coverage (UHC) goals of bringing TB treatment and care closer to patients’ communities [6]. CHS is defined as “*the set of local actors*,* relationships*,* and processes engaged in producing*,* advocating for*,* and supporting health in communities and households outside of*,* but existing in relationship to*,* formal health structures*” [11].

Literature suggests that many low- and middle-income countries (LMIC) that have invested in CHS experience gains in health status [12]. These gains are partly due to the ability of CHS to extend preventive and curative health services for infectious diseases such as TB into communities through integrated community-based delivery approaches [10, 13, 14]. CHS can be conceptualized through four main lenses; programmatic, relational, collective action, and critical perspectives [13, 15]. The ‘programmatic’ lens is concerned with CHS building blocks such as human resources, financing, technologies, and infrastructure [13, 15]. The ‘relational’ lens focuses on forms of co-production or co-creation in implementation processes [15, 26]. The ‘collective action’ lens is concerned with mechanisms and processes that enable actors in the CHS to mobilize and act collectively in implementation processes [13, 15]. The ‘critical perspectives’ lens examines what lies behind programmes, including uncovering the deeper social, political, historical, economic, and health systems forces that hinder change or programme performance [13, 15, 16].

Successful delivery of TB services through CHS can be maximized by using implementation strategies aimed at improving implementation effectiveness [17]. Implementation strategies comprise methods and techniques that ease adoption, implementation, maintenance, and scale-up of evidence-informed interventions [17]. Powell et al. defined 73 discrete implementation strategies that can support implementation [18]. These strategies form 9 main groups: engaging consumers, using evaluative and iterative strategies, changing infrastructure, adapting and tailoring services to the context, developing stakeholder interrelationships, using financial strategies, supporting clinicians, providing interactive assistance, and training and educating stakeholders [19]. Literature on implementation strategies has largely relied on evidence from high-resource settings. Documenting these CHS implementation strategies can enhance the adoption, acceptability, and sustainability of programmes for addressing public health problems such as MDR TB [20–23]. Further, such evidence can strengthen sustainability of the decentralization process in MDR-TB service delivery [20–24]. This research aimed to delineate specific implementation strategies guiding the decentralization of MDR TB services in Zambia.

## Methods

### Study setting

In 2014, the Ministry of Health reported that Copperbelt and Lusaka provinces recorded a higher prevalence of TB: 1,211 and 932 per 100,000 population [25]. In contrast, the Central, North-Western, and Western provinces had a prevalence between 400 and 600 per 100,000 population; and Eastern and Muchinga provinces had the lowest prevalence, about 200 TB cases per 100,000 population [25]. The proportions of patients with MDR TB among new and previous cases appear higher in densely populated regions [26]. In 2015, Ministry of Health reported 99 cases against an expected target of 275. The drivers for this high TB prevalence have been increasing population, increasing urbanization from overcrowding, exposure to silica dust in mining, and poverty levels [25]. In 2018 the Ministry of Health, through the National TB and Leprosy Programme, with the support of international partners, introduced and scaled up decentralized management of TB and MDR-TB cases from the two national hospitals to other hospitals. They aimed to overcome the challenges associated with centralised care and treatment, including prolonged hospital stay, delayed initiation of treatment, poor patient monitoring, and higher default rates [27].

### Study design

This study was part of the large comprehensive mixed-method project investigating the impact of decentralization of Multi-Drug-Resistant TB services in Zambia on uptake and adherence. It employed a qualitative case-study design to conduct an in-depth evaluation of implementation strategies applied in decentralized programmatic management, in the CHS context [28]. As shown below, the key aspects of the criteria for reporting qualitative research (COREQ) guidelines, such as description of the research team, study methods, findings, analysis, and interpretation, guided the study (See supplementary file).

### Data collection methods

Data were collected by teams of male and female researchers from the University of Zambia and Ministry of Health with postgraduate training in qualitative research as well as extensive experience in implementation science and MDR-TB. None of the team members had worked with the respondents. The team comprehensively developed rapport with the respondents before commencing data collection. All participants who were approached agreed to participate in the study. A total of 112 qualitative face-to-face interviews were conducted in private/ confidential spaces in their homes, workplaces and health facilities. The in-depth interviews lasted between 30 and 60 minutes.

Data collection methods were in-depth interviews (IDI) with healthcare workers [18], community health workers [17], patients [32], and caregivers [21] at selected healthcare sites. We also conducted key-informant interviews (KII) [24] with managers from health facilities, district, provincial, and national levels. The interview guides were piloted prior to data collection (See supplementary file). Involvement of the various stakeholders in the interviews allowed us to triangulate views across distinct levels of the health system.

Study participants were purposively sampled based on their role in MDR – TB treatment and management. In order to collect comprehensive data, we sampled respondents at various levels of the health system, as shown in Table 1.

**Table 1:** Participant information

Categories of respondents	Subtotal
Healthcare workers	18
Community health workers	17
Patients	32
Caregivers	21
Key informants (managers at facility, district, provincial and national levels)	24
Total	112

### Data analysis

All interviews were digitally recorded. Trained research assistants managed the recording and transcribed the data, with rigorous review by the co-authors to ensure consistency. We followed a thematic analysis approach: “a method for identifying, analysing and reporting patterns (themes) within data. It minimally organizes and describes a dataset in (rich) detail and goes further to interpret various aspects of the research topic” [29].

We used an inductive analysis approach to map data from the interview transcripts into the Expert Recommendations for Implementing Change (ERIC) compilation of strategies using the provided definitions and aligned the strategies to the categories proposed by Waltz et al. [18]. The field of implementation science has been growing rapidly over the past few years. In order to ensure consistency in reporting across studies, various taxonomies have been proposed to structure the description of key concepts. The ERIC framework provides 73 discrete strategies for operationalizing implementation strategies. It has been found applicable across a diverse range of settings, and we hope to contribute to expansion of its use [18].

The interview guides included a section on implementation process of MDR –TB. Because our primary interest was implementation of the decentralization processes, the interviews did not ask directly about the ERIC. A qualitative secondary analytical approach using a thematic coding framework allowed us to infer those strategies as shown in Table 2.

Ten coders independently conducted data management and analysis using NVIVO version 12, (QSR Australia). Once mapped, the categories were refined in the first three community health systems lenses: *programmatic*,* relational and collective action* (Table 2). Data saturation, at which no additional new information can be attained, was discussed during the data collection and analysis stages. Results and discussion sections integrated the fourth lens (critical perspectives) in form of barriers to delivering decentralized MDR –TB services. The mapping and categorizing of data were done by a team of researchers from the University of Zambia and Ministry of Health with experience in implementation science and MDR-TB. As part of the process, writing workshops allowed comprehensive discussions.

**Table 2:** Community health systems lenses and the corresponding ERIC clusters and discrete strategies

Community health systems lenses	ERIC clusters	Discrete strategies
Programmatic lens	Changing infrastructure	- Refurbishing and expanding health facilities to accommodate management of MDR TB
	Adapting and tailoring clinical and diagnostic services to the context	- Integrated outreach services - Enhanced referral system - Designating days for outreach services
	Training and educating health providers	- Providing ongoing training
	Using evaluative and iterative strategies to review program performance	- Developing and organizing quality monitoring systems - Regular audit and feedback processes
Relational lens	Providing interactive assistance	- Providing local technical assistance through clinical expert committees
	Supporting clinicians	- Developing health worker and community health work outreach teams - Zoning districts
Collective action lens	Engaging consumers	- Increasing demand by using community networks and events - Involving patients and family members - Intervening with patients to enhance uptake and adherence

### Ethics

Ethical clearance for this study was given by the University of Zambia Biomedical Research Ethics Committee (UNZABREC), Reference number 3003 − 2022. Further, permission to conduct the study was obtained from the Zambia National Health Research Authority and Ministry of Health. Prior to data collection, we obtained written informed consent from all participants. Participants were given an opportunity to seek clarification of the purpose of the research before choosing to participate. Also, participants were told that they had the right to withdraw or to stop the interview without any consequence.

### Findings

This section describes implementation strategies employed in managing MDR TB in Zambia. It is organized around the programmatic, relational, and collective action CHS lenses. Under each lens, Table 2 lists the main ERIC clusters and their discrete implementation strategies.

### Programmatic lens

Implementation strategies identified under the programmatic lens fell into four clusters: i) Changing infrastructure, (ii) Adapting and tailoring clinical and diagnostic services to the context, (iii) Training and educating health providers, and (iv) Using evaluative and iterative strategies to review program performance. Below, we explain the discrete strategies in each cluster. Quotations illustrate perspectives expressed in the interviews.

### Changing infrastructure

#### Refurbishing and expanding health facilities to accommodate management of MDR TB

Specific areas in refurbished health facilities were designated as MDR TB points of care. They ranged from entire refurbished buildings in Kabwe district to small wards in Mongu district and tents in Luanshya district. Both inpatient and outpatient services are provided (as bed space allows), and referrals are made only for complex cases. Implementing partner support was crucial to the refurbishing of such spaces.

“When we started, we were operating from a tent that was mounted somewhere in the hospital until we were given a shelter. So even when we started the decentralized system, we were still using a tent until maybe two years ago when we are given this structure. There were some renovations that were done in certain departments, and they were deemed fit to host TB services as those finally arrived here.” (001, KII, Coordinator).

In many parts of Zambia, where decentralization of MDR TB services had taken place, our study observed that patients had benefited a lot, by having services available at the nearest local health facility. This change was cost-effective: it reduced transportation expenses and distances covered, and fewer patients missed their appointment or review dates.

“…there is no need to spend a lot on transport as the hospitals are nearer, they treat you or you collect your drugs.” (003, IDI, patient).

Having MDR TB services closer to patients in the communities resulted in less congestion in health facilities. Because patients had many services nearby, they did not have to wait as long for results. This reduction in congestion allowed better interactions between patients and health workers.

“Also at least the patients go home early, at least they have a lot of rest, time for resting, also we are able to talk to patients one on one, because if they are a lot, you will be rushing so they can finish, but if they are few, at least a patient is able to open up to you if there is an inner problem that patient has?” (004, IDI, CHW).

The decentralized approach also enhanced interactions between patients and health workers because the health facilities were closer. The greater access also fostered stronger relationships between health workers and patients.

“Decentralized the system I think is, is the best. It’s the best because patients now have an interface with us, they know us because we are all locals in the province. So, you can even give them your number, they can call but I can imagine that time when it was in Lusaka.” (005, KII Coordinator).

However, absence of essential equipment, such as ECG machines, in certain facilities hindered health workers in effectively managing some patients. Additionally, drug shortages significantly impacted delivery of MDR TB services. These shortages were attributed to logistical and administrative challenges, including delays in drug procurement and challenges in transporting the drugs.

“(…) The DR TB drugs are more expensive, and we don’t want to just give facilities even if they don’t have patients. We only want to supply them with these a patient but that becomes a barrier what if I have a patient at short notice? So, it becomes a barrier on the part of the implementation.” (002, KII, Coordinator).

### Adapting and tailoring clinical and diagnostic services to the context

#### Integrated outreach services

Where microscopy had been the only diagnostic tool, GeneXpert machines and outreach trucks produced an increase in the number of TB cases identified. Where facilities lack GeneXpert, alternative equipment (such as fluorescent microscopes, digital X-ray machines, or LAM tests) is often not readily available. Many of these facilities only have light microscopes or face shortages of essential reagents. Deployment of outreach trucks equipped with diagnostic equipment and supplies, including X-rays, improved identification and treatment of active cases, enhanced patients’ access to care, reduced costs, and led to improved health outcomes. The trucks also significantly reduced the distances that patients had to travel, particularly when the sites had full diagnostic and treatment capacity.

“We target those areas with active case finding where we go and do public announcement with PA system, public sensitization then the patients will also go with the mobile TB truck because we have two aah mobile TB trucks as a province one which we were given by eradicating TB so the mobile TB truck has got the GeneXpert machine on it, one has got 16 modules then the other one has a four modules so what we do we have a team, we form teams of clinician to be doing the screening we have lab techs on that.” (006, KII Coordinator).

#### Enhanced sample referral system

Where samples still required transportation to Lusaka, most districts used a courier system such as *FedEx* and *Zampost*. This system enhanced diagnostic capacity in the newly established sites by linking smaller clinics to larger health facilities. In remote districts, where facilities lacked proper diagnostic tools and could not use a courier system, transportation of specimens was integrated into other health programmes, such as HIV Viral load testing.

“Laboratory but this time around the ministry of health has initiated a way that the samples are transported by Zampost (Postal Services Company) so when we prepare the samples in the cooler boxes everything, we just call the staff at the Zampost to say the samples are ready to go then they send a motorbike rider who comes and takes them. I think the movement of the samples has been very good compared to the gap which was there that we needed to be moving the samples physically to Lusaka.” (007, KII, Provider).

#### Designating days for outreach services

Further adaptations for improving treatment included some facilities’ designating days on which they could attend to MDR TB patients. This marked a significant shift from the pre-decentralization era, when patients who missed their appointment dates at the University Teaching Hospital had to wait a month before they could see a provider again. Patients had more flexibility in rescheduling their review dates. Their local facilities now managed relatively fewer MDR-TB cases, in contrast to the previously more congested central facility.

“If they miss their appointment they will come if they ask for a different date will arrange for them to be seen by the doctor or call the doctor to say this patient didn’t come on the actual date but he has come today he said he wasn’t around or she wasn’t around there and then they will be seen, like UTH they never used to do that, I don’t think so. Because when they miss an appointment maybe someone will say no come on this period in time.”(008, KII, CHW).

However, lack of functional ambulances in some districts also hindered transportation of patients to health facilities that had adequate services. This transportation challenge delayed access to care and, in some instances, contributed to the spread of the disease.

“On care and treatment, I think that is where we have a bit of a challenge, for example I would say if a patient has been diagnosed from a facility, there is no readily available vehicle to pick them and take them to Solwezi general hospital so there are challenges when it comes to transport, so there will be delayed initiation.” (009, KII, coordinator).

Despite having courier systems, healthcare providers acknowledged the resource-intensive nature of this process, especially in scenarios with a very limited number of patients. Providers faced a unique challenge in balancing the efficiency of courier services against the sporadic and often low volume of TB specimens requiring transportation. As one provider noted:

“So that poses us a challenge because we do this once every month and at the same date when we do, either the same day or the next day, we also have to go get the specimen that we collected from them and take to TDRC. So, if a patient comes as a loner, it becomes difficult to get that specimen back to DRC.” (010, KII, Coordinator).

### Training and educating health providers

#### Providing ongoing training

Community health workers and nurses were trained in Directly Observed Therapy (DOT) to support MDR TB treatment. The aim was to improve adherence to TB medication regimens by regularly monitoring patients as they take their TB drugs. Training was also provided on how to conduct community sensitization and contact tracing.

“They had also trained them to follow patients in their homes, then it was very easy because they were also on the DOT’s program because they would make sure that patient takes drugs …. if they have not taken, they would come to report.” (011, KII, Provider).

Community health workers explained how this training enhanced their ability to effectively monitor patients’ adherence to treatment. The monitoring process entailed visiting the patients at both homes and workplaces. This proactive approach played a crucial role in improving patients’ uptake of drugs.

“So, because of this decentralization it’s easy for us to monitor since these clients are closer to us. They are close to our facilities, and also close to our TB treatment supporters. So the monitoring of these patients is better. Many are able to adhere to treatment.” (012, KII, Coordinator).

However, the training often overlooked those working in hospital areas where encounters with MDR TB patients are more frequent, such as outpatient departments and among nutritionists. The training content, however, did not explicitly cover providing service for populations with unique needs, such as children. Some districts addressed this gap through supplementary training supported by external partners.

“I think in 2020 we had some children who needed treatment there, and were presumed to have MDR TB sometime in January. And the worst part was that that time, only few were trained in MDR TB management for little one. So what we have gone for training was basically for adults, so it came as a challenge. But I knew with time it was phased (training was planned), CIDRZ came into train people in childhood MDR TB management.” (014, KII, Coordinator).

### Using evaluative and iterative strategies to review program performance

#### Developing and organising quality monitoring systems

To ensure delivery of a high quality of care, a key function of the decentralization process was establishing a surveillance system for MDR TB. Appropriate registers such as the MDR TB surveillance registers, which fed into existing monitoring and evaluation systems, were introduced to ease collection of data. The registers were aligned to other registers such as the TB treatment register, lab registers, and contact tracing registers. Patients were also given treatment cards. Though clinic meetings and audits were conducted, they were felt to place little emphasis on MDR TB, compared with presumptive TB. Provincial coordinators pointed out that they were able to generate quarterly reports to gauge the performance of the programme. One recommendation suggested to improve the surveillance system is to transition the paper-based registers to an electronic system.

“I think we need to go electronic, we need to use something like smart care, maybe we have been reluctant but I think it is something that can help us deliver the service better, why I am saying so is that if you decentralize and there is someone at the facility who doesn’t know how to manage the patient, sometimes I would just log in and see what is going on and help, so in short I think electronic/telemedicine should be in incorporated and it will help in the decentralization, so we need think ahead that we are in an era of technology.” (018, KII, Coordinator).

#### Regular audit and feedback processes

Weekly TB situation rooms provided an opportunity to review the performance of the decentralized MDR TB services and identify strategies to improve them. Quarterly clinical expert committee meetings reviewed difficult cases and provided technical support on the best patient management strategies.

“We also hold the quarterly clinical expert committee meetings where we review like patients like difficult patients so like each district has been given a chance to present on a difficult case that they have had in that quarter both for MDR TB and drug susceptible so in that platform we build capacity and we have a team experts that now advice on how that patient can be managed and also aah we have really improved in term of treatment outcome for DR patients.” (019, KII, Coordinator).

### Relational lens

Implementation strategies under the relational lens fell into two clusters: i) Providing interactive assistance and (ii) Supporting clinicians. Discrete strategies under each cluster strategy are explained below.

### Providing interactive assistance

#### Providing local technical assistance through clinical expert committees

Various systems created spaces where health workers could interact, discuss cases, and mutually support one another in delivering MDR TB services. Clinical expert committees, including physicians, nurses, and other healthcare workers, were established as a key component in managing MDR TB. Their primary function was to review complicated MDR TB cases. By using the collective ability of its members, the committee could provide more effective treatment strategies. Availability of committees significantly enhanced quality of treatment. The frequency of their meetings depended on the reported complications. This adaptive approach allowed prompt intervention in urgent or severe cases, while also ensuring efficient use of resources during periods with fewer complex cases.

“Besides that, I think as a district we also participated ahh we were part of what was being called the clinical expert committee, that clinical expert committee I think there are a number of people, there are physicians, there are nurses and other healthcare workers.” (020, KII, Coordinator).

During national meetings, well-performing provinces served as examples of best practices, to create an incentive for providers to continually review their activities with an overall goal of improvement.

“Remember we never used to have the structures we have now, whenever you have the national expert committee, a provincial expert committee, we never used to have those structures, but when we started decentralizing, we started the trainings and put in place the structure such that we have got a provincial CEC now where we are making consultations.” (010, KII, Provincial TB Coordinator ).

However, the ability of provincial expert committees to travel across districts, providing technical assistance and facilitating implementation of decentralized services, depended on availability of resources. This constraint resulted in less frequent visits in recent years.

“There was an activity where we were doing whether we had to do peer-to-peer data quality audits …and have also cut on the economic cost to the client as well.” (021, KII, Coordinator).

### Supporting clinicians

#### Developing health worker and community health work outreach teams

To reduce the spread of the disease, trained teams and community-based volunteers conducted contact tracing among family members, friends, and neighbors of identified cases in the study districts. Contacts at hospitals, particularly bedside attendants, were also tested. Further, active case detection strategies focused on TB hotspots by conducting outreach programmes using specially equipped TB Trucks. The outreach work was a collaborative effort between community health workers (CHWs) and health workers. Providers pointed out that this strategy was effective in finding cases, particularly among populations such as business people who would have otherwise been missed by passive facility-based case-finding strategies.

“We form teams of clinicians to be doing the screening, we have lab techs on that aah on that mobile aah mobile vehicles the lab tech that will doing the sputum using the gene expert then we also have the nurses as well as CBVs that help us in terms of aah sputum collection as well as mobilizing and going door to door doing sensitization so each time, we conduct active case finding in the community.” (022, KII, Coordinator).

Involvement of CHWs, such as TB promoters attached to local health facilities, helped in scaling up contact tracing because they were able to target immediate contacts or neighbors of TB patients. CHWs played many roles in MDR TB, including helping clients with transportation, translation services, and psychosocial support. Further, the CHWs supplied valuable information on TB prevention, such as promoting regular opening of windows to reduce the spread of the disease.

“Then those who are already patients, sometimes we go in the communities for contact tracing. For example, for a patient who is on TB drugs we talk to that patient. Can we come to your place because TB of the sputum is infectious, so we follow them in the community to do contact tracing, we also go in the wards to do contact tracing especially for the ‘bed-siders’.” (024, KII, CHW).

#### Zoning districts

To ensure that clinician outreach teams reached even the most remote communities, the district was divided into zones, and a schedule was created for clinicians to visit patients in these designated zones. This approach helped to reduce costs and other travel-related barriers, thus improving access to services for all people including the most vulnerable.

“We have come up with the schedule where we have zoned the district into four zones and we have picked the high volume facilities where we are reviewing our DR patients because we looked at it since we don’t have resources uhm to give to all the patients as in form of transport money so it is cheaper to move the clinicians like following the patients to those facilities which is nearest where they are staying so that at least the patients can be able to come and be reviewed.”(023, KII, Coordinator).

Contact tracing and follow-up have produced noticeable improvements in the ability to retain patients in the treatment and care program.

“Yes, if you have not finished taking your medication, they make follow ups. You don’t have to stop taking your medicine because once you complete it you will get better. They don’t tolerate stopping medication, they even follow you home.” (025, IDI, ALE-TB Patient).

Conversely, challenges posed by inadequate phone network coverage or connectivity also had a significant impact on dissemination of MDR TB information. In areas with weak or unreliable network signals, TB promoters met considerable obstacles in their efforts to maintain regular patient follow-up. The struggle to establish consistent and effective communication channels in these regions underscored the urgency of improving infrastructure and technological support to ensure the continuous flow of critical health-related information.

“I have patients in Lukulu in an area that is cut off. Healthcare workers can’t reach there, So I’m worried about those patients and there’s no network.” (026, KII, Coordinator).

Further, the COVID-19 pandemic had a negative effect on feasibility of conducting directly observed therapy. Because of safety precautions, patient visits were scaled down to alternate days instead of daily. Additionally, facility staff who contracted COVID-19 were unable to deliver essential services.

“Well, I think there was a huge drop in OPD attendances. … people were avoiding coming to the hospital for two reasons…., they did not like the procedure of the nasal swab. …Then secondly, the stigma… once you are COVID-positive. When you fell sick, you would be isolated. Your patient and their family could not see them at the height of the second wave. So, there was that fear.” (027, KII, Coordinator).

### Collective action

The cluster implementation strategy, engaging consumers, was actualized through three discrete strategies.

### Engaging consumers

#### Increasing demand by using community networks and events

For engaging or raising awareness about MDR TB patients, communication of key MDR TB health messages used channels such as posters in health facilities and radio jingles. To maximize demand for services, CHWs integrated MDR TB services into other outreach activities, such as screening for malaria and community sensitization activities on other diseases. Further, CHWs used public spaces such as churches and schools to deliver information on MDR TB. That strategy allowed CHWs to reach out to as many people as possible within the brief period.

“Yes, we used to involve them for the reasons that; if we have an issue of contact tracing, we never used to segregate that it is only MDR TB patients to be followed, we were also following the surrounding houses those that are near and even them at the gathering place. Sometimes we used to involve churches so that everyone could have an idea that indeed TB is there.” (028, IDI, CHW).

CHWs also used communal events to deliver information on MDR TB. Major commemorations such as World TB Day provided opportunities to conduct TB sensitization and raise awareness.

“We have integrated the information education and communication like the IEC at facility level especially like a month like this month like a month of march you know that march is aah a world TB day march so we have intensified like heightened a lot on TB sensitization and awareness so we have also integrated like ah TB sensitization in programs like school health services like during GMPs we are also talking about TB also the debates now like we currently have the debates running like in Kitwe and Lufwanyama so that we also use that platform to sensitize on TB, yes.” (029, KII, Coordinator).

However, translation of the messages into the local languages affected communication of the information. For example, multi-drug-resistant tuberculosis was sometimes misinterpreted as TB that cannot be cured. This misunderstanding led to a reduction in the acceptability of the MDR TB services.

“Maybe one day the term drug resistant may need to be revised because if you literally translate these drug resistant it simply means this TB which cannot be cured but what it means to professionals simply means that drug resistant this is TB which is resistant to face blind drugs face blind but if you just go in the community and say drug resistant, for them they know that this is TB which cannot be cured …we may come up with the better term.” (031, KII, Coordinator).

Societal myths and misconceptions also led some patients to not seek MDR TB services. Some patients and community members, for example, believed that people who have TB are also infected with HIV. The association of TB with HIV made some patients shun TB clinics. Others believed that those who were on medication could still infect others with TB.

“It’s TB we are talking about here and we hear rumors that the disease is contagious but then I had no choice but to convince myself that he will be okay since he is already on medication and receiving treatment. Most people say those with TB are also HIV patients.” (032, IDI, Caregiver).

#### Involving patients and family members

Further, families were identified as being an essential support system for patients. When the services were centralized, long distances limited involvement of families. With services nearby, in districts such as Ndola, neighbors could be also involved in collecting sputum samples.

“What was happening then, and we lost quite a number of patients because everybody that was there it was being taken to Lusaka and there was no social network with family and friends in Lusaka.” (034, KII, Coordinator).

With MDRTB treatment now accessible at local healthcare facilities and critically ill patients being admitted there, it has become more convenient for them to receive emotional and moral support from their friends and relatives, all without any added costs. Accessing MDR TB services in local facilities also made it easy for caregivers to nurse the patient while simultaneously managing their other responsibilities.

**“**Actually, we are happy that she is receiving the treatment from here because if it was the issue of going to Lusaka it means for one to visit her, they need to prepare transport money but the way we are it’s fine she just goes for review and sometimes we can collect medicine for her.” (035, IDI, Caregiver).

Community health workers reported that decentralization had enabled them to make frequent visits and thus strengthened social bonds with their patients. Further, providing medication also promoted social bonds.

“As they were seen that you are frequently going there, we kind of created a family and immediately they see you they will say my mother I have already taken the medication.” (036, CHW).

Although community networks and events were supportive, social stigmatization affected implementation. Cases of TB stigma were also reported among family and friends. Some family members distanced themselves from MDR TB patients, whom they viewed as outcasts.

“Am saying the community looked at someone with MDR TB like an outcast.” (039, KII, Coordnator).

#### Intervening with patients to enhance uptake and adherence

Initially, patients received transport refunds to come for clinical reviews, and also food supplements. The National TB policy states that each patient ought to receive a full basket with various foods, including 2 kg of beans, 5 L of cooking oil, 2 kg of sugar, and 1 kg of soya flour. Yet responses to food baskets varied. Provinces with active partner support were able to continue providing some food, even though it was a diminished food basket with a handful of items. In Lusaka, for instance, patients were receiving only soya flour, whereas some districts provided no food at all.

“We also offer supplementary nutrition food to people who are actually, to people who actually in care of TB, yah, things like soya, herbs, some eggs and when we have enough even transport for them to pick, to pick up the drugs, yah, so, basically its one area that we actually, one area that we want people to know and come because we have all these things at their dispose.” (015, KII, Coordinator).

This support, however, later came to a halt in many provinces after partner support ended. In Copperbelt province, for instance, patients used to receive a K250 (approximately $10) transport refund, but that stopped once partner support ended in 2021. Both providers and patients pointed out that patients tended to stop taking medication midway because of challenges related to provision of the food basket and transport from the health facility.

“So now people stopped coming because they are taking medication without any food assistance hence others stopped coming.” (016, IDI, Patient).

Health workers mentioned that poor nutrition, because of limited capacity to buy food, also affected adherence to treatment and hence healing. They noted that this situation was complicated by the end of the nutritional support programme in the health facilities.

“.. sometimes you find that you run out of food, then you take those drugs, some medicines it is required to say when you take you eat something. But when you don’t have food, it is very difficult.” (017, IDI, Patient).

## Discussion

The study aimed to document discrete implementation strategies needed to effectively deliver decentralized MDR TB services in community health systems in low-resource settings. It contributes to enhancing community-based implementation research by contextualizing the ERIC compilation of implementation strategies in decentralized CHS service delivery [18, 19]. This contextualization using the CHS programmatic, collective action, and relational lenses adds value to community-based implementation strategies in many ways.

First, the programmatic lens outlines discrete implementation strategies for strengthening CHS hardware components in delivering decentralized MDR TB services. These strategies, which include changes to health facilities, training, and increased availability of equipment and data/information in communities, provide structures and information for community outreach activities and for relational and collective-action-related implementation strategies. For example, programmatic lens decentralization strategies, such as refurbishing and expanding many health facilities in rural areas, training health workers including community health workers, using mobile trucks fitted with equipment and other medical supplies, eased delivery of quality MDR-TB services. These strategies contributed to improved utilization by enabling people to have a variety of quality services in their communities, as other studies have reported [30–34]. Providing services in the communities is also often preferred, as it reduces distances to health facilities and other related costs [11]. We thus agree that, through leveraging various community resources, CHS can promote uptake of various health services and possibly advance population well-being in attaining universal health coverage [7, 35–37].

Second, relational implementation strategies are vital in delivering decentralized MDR TB services, as they facilitate strengthening linkages between health facilities and communities. One of the main relational strategies is the development of health worker and community health worker outreach teams. Involvement of community health workers also helped in increasing coverage and use of MDR –TB services. Acceptability improved because CHWs are recognized, trusted, and respected members of the community [38]. Developing such teams could enhance implementation of MDR-TB services by providing opportunities to accommodate diverse actors, interests, and expressions of power that characterize the CHS [22, 35, 39]. Further, relational approaches can support delivery of services by promoting shared communication, understanding of implementation problems, and increased commitments [40]. This collaborative approach can trigger better adaptation and acceptability of services through promoting local ownership and legitimacy of implementation strategies [22, 29, 30]. Use of spaces for consensus building during the implementation process, such as clinical expert committees, in which clinicians discuss complex cases, can also facilitate adoption of services by facilitating or enabling co-production and co-learning during service delivery [15, 23, 29].

Third, collective action implementation strategies, such as use of community networks, structures, and events to deliver and disseminate information, also promoted acceptability, penetration, and sustainability of decentralized MDR TB services at the community level. Use of community-based resources supports implementation processes by enhancing relevance and legitimacy of the services [41, 42]. In addition, these strategies are vital in community-based implementation, as there are “*mechanisms and processes which enable actors in the CHS to mobilize*,* collaborate and act collectively on health*” [13]. Importantly, the strategies promote sustainability and penetration of health programs through widening participation and accountability as well as promote trust in health services [21, 39].

However, health systems and socioeconomic barriers affected implementation of decentralized programmatic management of multidrug-resistant tuberculosis. Health systems barriers included lack equipment such as functional ambulances. Socio-cultural barriers were termination of nutritional support, as well as local myths and stigma relating to TB. To address these barriers, it is important to integrate a critical perspective approach *(critical lens)* in the implementation process in order to regularly identify or uncover deeper social and health systems forces that affect implementation [11]. By uncovering structural barriers, a critical perspective might help to trigger collective action aimed at promoting positive change in programme implementation processes [11].

### Limitations and strengths of the study

One limitation of this study is the absence of implementing partners that support the implementation of MDR-TB in the sample. This absence limited the scope of the insights shaping decentralisation to largely stakeholders from the Ministry of Health, caregivers and patients. Another limitation is that the interview guide did not directly integrate the ERIC strategies, but we only inferred to the strategies from the responses provided during the interviews on implementation of the decentralization process. This lack of inclusion could have deprived us of an opportunity to gain additional insights on contextual strategies that shaped the implementation. Despite these limitations, conducting inclusive interviews with stakeholders at different levels of the health system (community, district, provincial, and national levels) provided useful information to facilitate a comprehensive understanding and implementation MDR TB services in Zambia.

## Conclusion

The study builds on the ERIC compilation of implementation strategies by documenting CHS contextual discrete strategies applied in implementing decentralized programmatic management of MDR TB. The programmatic CHS-lens-related implementation strategies included refurbishing and expanding health facilities to accommodate management of MDR TB, adapting and tailoring clinical and diagnostic services to the context, and training and educating health providers. Relational CHS lens strategies included providing interactive spaces through clinical expert committees and developing health worker and community health worker outreach teams. Collective action CHS strategies consisted of community-driven integrated approaches to engaging MDR-TB patients.

In addition to mapping the ERIC strategies, the study has several policy and program implications. For example, it further contributes to community-based policy and implementation research by documenting relevant discrete relational and collective action strategies, namely, developing health worker and community health worker outreach, as well as increasing demand using community networks and events, respectively. Mapping CHS implementation strategies could promote implementation processes by helping experts appreciate the complexity of CHS strategies -- as being embedded in both health facilities and communities -- and develop program and policies that adequately integrate both formal and community aspects of the health system. Thus, to work effectively, the programmatic lens strategies need to be supported or integrated within strategies that promote meaningful community engagement (relational lens) and should be attuned and responsive to strategies that promote forms of community mobilization (collective action lens), while being cognizant of wider social and systems forces or barriers that affect the implementation process (critical lens).

## Electronic supplementary material

Below is the link to the electronic supplementary material.

Supplementary Material 1

Supplementary Material 2

## Data Availability

The datasets developed and/or analysed are available from the corresponding author on reasonable request.
