# Supplementary material for: Implementation strategies for decentralized management of multidrug-resistant tuberculosis: insights from community health systems in Zambia
Source: Arch Public Health. 2024 Sep 14;82:157. doi: 10.1186/s13690-024-01384-4 (PMC11401366; doi:10.1186/s13690-024-01384-4)
Supplement: Supplementary file 1 — Supplementary Material 1 [file 13690_2024_1384_MOESM1_ESM.docx]

Supplementary File 1: COREQ checklist

Consolidated criteria for reporting qualitative studies (COREQ): 32-item checklist

**Manuscript title:** Implementation strategies for Decentralized Management of Multidrug-Resistant Tuberculosis:

Insights from Community Health Systems in Zambia

Developed from: Tong A, Sainsbury P, Craig J. Consolidated criteria for reporting qualitative research (COREQ):

a 32-item checklist for interviews and focus groups. International Journal for Quality in Health Care. 2007.

Volume 19, Number 6: pp. 349 – 357

| **Item No** | | **Guide Questions/Description** | **Reported on Page #** |  |
| --- | --- | --- | --- | --- |
| **Domain 1: Research team and reflexivity** | | | |  |
| **Personal Characteristics** | | | |  |
| 1. Interviewer/ facilitator | | Which author/s conducted the interview or focus group?  *All the authors contributed to the design of the study including the data collection tools and collecting data. JMZ, PM, HH, MPC, MM, TM, NS and BH participated in analysing the results of the study.* | Pg 23 |  |
| 2. Credentials | | What were the researcher’s credentials? E.g., PhD, MD  *Data were collected by team of male and female researchers from the University of Zambia and Ministry of Health with postgraduate training in qualitative research as well as extensive experience in implementation science and MDR-TB.* | Pg 6 |  |
| 3. Occupation | | What was their occupation at the time of the study?  *Data were collected by team of male and female researchers from the University of Zambia and Ministry of Health with postgraduate training in qualitative research as well as extensive experience in implementation science and MDR-TB.* | Pg 6 |  |
| 4. Gender | | Was the researcher male or female?  *Data were collected by team of male and female researchers from the University of Zambia and Ministry of Health with postgraduate training in qualitative research as well as extensive experience in implementation science and MDR-TB.* | Pg 6 |  |
| 5. Experience and training | | What experience or training did the researcher have?  *Data were collected by team of male and female researchers from the University of Zambia and Ministry of Health with postgraduate training in qualitative research as well as extensive experience in implementation science and MDR-TB.* | Pg 6 |  |
| **Relationship with participants** | | | |  |
| 6. Relationship established | | Was a relationship established prior to study commencement?  *None of the data collection team members had worked with the respondents*. | Pg 6 |  |
| 7. Participant knowledge of the interviewer | | What did the participants know about the researcher? e.g. personal goals, reasons for doing the research?  *None of the data collection team members had worked with the respondents*. | Pg 6 |  |
| 8. Interviewer characteristics | | What characteristics were reported about the interviewer/facilitator? e.g. Bias, assumptions, reasons and interests in the research topic  *Data were collected by team of male and female researchers from the University of Zambia and Ministry of Health with postgraduate training in qualitative research as well as extensive experience in implementation science and MDR-TB.* | Pg 6 |  |
| **Domain 2: study design** | | |  |  |
| **Theoretical framework** | | |  |  |
| 9. Methodological orientation and Theory | What methodological orientation was stated to underpin the study? e.g. grounded theory, discourse analysis, ethnography, phenomenology, content analysis  *It employed a qualitative case-study design to conduct an in-depth evaluation of implementation strategies applied in decentralized programmatic management of MDR-TB, in the CHS context of Zambia (28).* | Pg 6 |  |  |
| **Participant selection** | | |  |  |
| 10. Sampling | How were participants selected? e.g., purposive, convenience, consecutive, snowball  *Study participants were purposively sampled based on their role in MDR – TB treatment and management.* | Pg 7 |  |  |
| 11. Method of approach | How were participants approached? e.g., face-to-face, telephone, mail, email.  *A total of 112 qualitative face-to-face interviews were conducted to delineate the specific implementation strategies employed in managing MDR TB in community health systems in Zambia.* | Pg 6 |  |  |
| 12. Sample size | How many participants were in the study?  *A total of 112 qualitative face-to-face interviews were conducted to delineate the specific implementation strategies employed in managing MDR TB in community health systems in Zambia.* | Pg 6 |  |  |
| 13. Non-participation Setting | How many people refused to participate or dropped out? Reasons?  *All the participants who were approached to participate in the study agreed to do so.* | Pg 7 |  |  |
| 14. Setting of data collection | Where was the data collected? e.g., home, clinic, workplace  *Interviews were done in private/ confidential spaces within their homes, work places and health facilities.* | Pg 6 |  |  |
| 15. Presence of nonparticipants | Was anyone else present besides the participants and researchers? | N/A |  |  |
| 16. Description of sample | What are the important characteristics of the sample? e.g. demographic data, date  *Table 1: Participant information* | Pg 7 |  |  |
| **Data collection** | | |  | No |
| 17. Interview guide | Were questions, prompts, and guides provided by the authors? Was it pilot tested?  *The interview guides were piloted before data collection could commence (See supplementary file).* | Pg 7 |  |  |
| 18. Repeat interviews | Were repeat interviews carried out? If yes, how many? | N/A |  |  |
| 19. Audio/visual recording | Did the research use audio or visual recording to collect the data?  *All interviews were digitally recorded. Trained research assistants managed the data recording and transcription, with rigorous review by the co-authors to ensure consistency.* | Pg. 7 |  |  |
| 20. Field notes | Were field notes made during and/or after the interview or focus group?  *Field notes made during interviews.* | Pg.6 |  |  |
| 21. Duration | What was the duration of the interviews or focus group?  *The interviews lasted between 30 and 60 minutes. Field notes made during interviews*. | Pg.6 |  |  |
| 22. Data saturation | Was data saturation discussed?  *Data saturation, which is the stage when no additional new information can be attained, was discussed during the data collection and analysis stages.* | Pg 8 |  |  |
| 23. Transcripts returned | Were transcripts returned to participants for comment and/or correction? | N/A |  |  |
| **Domain 3: analysis and findings** | | |  |  |
| **Data analysis** | | |  |  |
| 24. Number of data coders | How many data coders coded the data?  *Data management and analysis was conducted independently by 10 coders using NVIVO version 12, (QSR Australia).* | Pg 8 |  |  |
| 25. Description of the coding tree | Did the authors provide a description of the coding tree?  *Once mapped, the categories were further categorized in the first three community health systems lenses: programmatic, relational and collective action (Table1).* | Pg 8 |  |  |
| 26. Derivation of themes | Were themes identified in advance or derived from the data?  *We thus deductively/ inductively assigned strategies to the ERIC taxonomy as shown in Table 2.* | Pg 8 |  |  |
| 27. Software | What software, if applicable, was used to manage the data?  *Data management and analysis was conducted independently by 10 coders using NVIVO version 12, (QSR Australia).* | Pg 8 |  |  |
| 28. Participant checking | Did participants provide feedback on the findings? | N/A |  |  |
| **Reporting** | | |  |  |
| 29. Quotations presented | Were participant quotations presented to illustrate the themes/findings? Was each quotation identified? e.g., participant number | Pg 9-19 |  |  |
| 30. Data and findings consistent | Was there consistency between the data presented and the findings? | Pg 9-19 |  |  |
| 31. Clarity of major themes | Were major themes clearly presented in the findings? | Pg 9-19 |  |  |
| 32. Clarity of minor themes | Is there a description of diverse cases or a discussion of minor themes? | Pg 20-21 |  |  |
