# Supplementary material for: Implementation strategies for decentralized management of multidrug-resistant tuberculosis: insights from community health systems in Zambia
Source: Arch Public Health. 2024 Sep 14;82:157. doi: 10.1186/s13690-024-01384-4 (PMC11401366; doi:10.1186/s13690-024-01384-4)
Supplement: Supplementary file 2 — Supplementary Material 2 [file 13690_2024_1384_MOESM2_ESM.docx]

**Supplementary File 2:**

**Key Informant interview guide**

**Introduction**

1. Please tell me about yourself and your professional background?
   1. Probe for Role, length of experience implementing TB services.
   2. How have you been involved in the implementation of programmatic management of MDR TB?
2. How would you describe the knowledge and understanding of TB and Multi Drug Resistant TB in Zambia/district/Province?
   1. Is it easy for patients to seek diagnosis? Probe for barriers and facilitators
   2. Is it easy for patients to seek care and treatment? Probe for barriers and facilitators

**Decentralization and implementation processes**

**Exploration**

1. Previously MDR TB services were provided centrally? Could you describe how services were arranged before decentralization?
2. What prompted the need for decentralization?
   1. What were the main drivers for the decentralization of these services?
   2. What did you hope the decentralization process would result in?
   3. Was this the first time that your organization contemplated the decentralization of MDR TB services? Probe for a needs assessment.
   4. Who spearheaded the decentralization process?
3. What strategies were used to ensure that the decentralization process was compatible to:-
   1. Existing organizational structures, workflow and systems
   2. The beliefs, values and roles of implementers/providers
   3. The beliefs and values of the local communities, care providers and patients
4. The decentralization process has been conducted in a phased manner, what were some of the considerations regarding the locations that were prioritized? Please describe them

**Preparation**

1. Prior to the beginning of the implementation of decentralized MDR TB services, what are some of the preparatory measures that were taken in Zambia/district/Province?
   1. Probe for assessments of available resources, training of providers and CHWs, hiring of new staff, tailoring intervention strategies to local needs, purchasing equipment and supplies, establishment of M& E systems, pilot initiatives or awareness raising.
   2. Which actors were key in seeing to it that preparations were undertaken smoothly?

**Implementation**

1. How has the decentralization process unfolded in Zambia/district/Province? How did it start? How did the different stakeholders respond to it?
2. What some of the actors/ department involved the decentralizations process of the program province/ district? (Probe for NGOs, churches, traditional leaders etc)
   1. How were the different stakeholders engaged to be a part of the implementation?
   2. How do they work together to implement decentralized TB services?
   3. What are the strengths and weaknesses of the collaboration?
3. How are the coordination processes for the delivery of decentralized Drug-Resistant TB Services across partners / stakeholders done?
   1. What has worked well with this stakeholder coordination/ partnerships?
   2. What are gaps in the partnership/ coordination?
   3. How do issues of power and collective action affect the collaboration? Probe for trust, relationship building?
4. Are there any stakeholders that have been left? If yes, why? What could have done to bring them on board?
5. What can be done to improve the collaboration of stakeholders in implementing decentralization of Drug-Resistant TB Services?
6. How complex do the different stakeholders including patients find the decentralized services to be?
   1. Do you feel as though the providers are in a good position to implement the services based on the strategies put in place before initial implementation?
7. What type of resources have been necessary for the implementation of decentralized MDR TB services? Probe for space, equipment, time, staff, program materials and source of resources.
8. Have the providers been implementing the decentralized services as they were intended?
   1. Why do you feel this is the case?
   2. What challenges are they facing that affect how well they implement the services as planned?
9. What are some of the policies which are relevant and that guide or support implementation MDR TB (list them)?
   1. How do these policies affect the implementation of TB-MDR?
      - How do the policies support implementation of TB-MDR?
      - How do the policies hinder implementation of TB-MDR?
      - How can the policy environment be improved to better support the implementation of TB-MDR?
10. Have any changes been made to the decentralization policy and its implementation?
    1. What drove the need for these changes?
    2. How did the staff know which changes were the most appropriate?
11. What are some of the barriers and facilitators to the implementation of decentralized MDR TB services?

**Effectiveness of decentralization on MDR process, treatment and implementation outcomes**

1. Are you able to tell whether the decentralization is working? How can you tell?
   1. Has the decentralization process resulted in the recruitment of more patients? What has been the effect of decentralizing MDR-TB on the health facilities that used be treatment centers?
   2. What are some of the positive consequences that you have observed
   3. What are some of the negative consequences that you have observed
   4. What have patients and care providers said about the services since decentralization? Any feedback?
2. How would you describe patient experiences in accessing MDR-TB care and treatment now that services have been decentralized?
   1. At the treatment center? Probe for barriers and facilitators to accessing services.
   2. Within the community? Probe for barriers and facilitators to accessing services.
3. To what extent are patients adhering to treatment under the decentralized mode?
   1. Probe for barriers and facilitators to treatment adherence
4. Are the groups of patients for whom the decentralization has had less impact than others? Probe for specific categories and reasons why that is.

**Success and challenges of decentralization**

1. How has COVID-19 affected the implementation of decentralized MDR-TB services in Zambia/Province/district/facility? (To help assess the resilience of the program)
2. What are some of the key successes and challenges to the implementation of decentralized MDR TB services in Zambia/district/Province?

**Sustainment**

1. Are there any contextual changes coming up in the foreseeable future that you think may have an impact on the delivery of decentralized services?
2. What is the likelihood that the implementation of the decentralized services is going to continue in the long term?
   1. Do you feel as though it will be integrated in to existing systems?

What would be needed for the services to be delivered in the long term?

**Recommendations**

1. Based on your experiences, do you have any recommendations on how to improve the implementation of drug resistant TB services?

**Provider/ CHW interview guide**

**Introduction**

1. Please tell me about yourself and your background?
   1. Probe for Role, length of experience implementing TB services.
2. How would you describe the knowledge and understanding of TB and multi drug resistant TB within your facility/community?
   1. Is it easy for patients to seek diagnosis? Probe for barriers and facilitators
   2. Is it easy for patients to seek care and treatment? Probe for barriers and facilitators
3. When did you first learn about the decentralization of MDR TB services? From whom?
   1. When you first heard about the model you would be implementing what were your initial thoughts?
   2. Was there any initial resistance from you or your colleagues?

**Decentralization and implementation Processes**

1. How is the delivery of TB services currently structured within your facility/community?
   1. What services are being provided?
   2. Is this the same structure that is used to provide services for multi drug resistant TB? Probe for treatment centres, community outreach services.
   3. Who is responsible for implementing these services?
2. Has this always been the way multi drug resistant TB have been implemented within your facility/community?
   1. What are some of the major changes that have taken place over the past few years in how multi drug resistant TB services are delivered? Please describe them.

**Preparation**

1. Prior to the beginning of the implementation of decentralized MDR TB services, what are some of the preparatory measures that were taken in your facility/community?
   1. Probe for assessments of available resources, training of providers and CHWs, hiring of new staff, tailoring intervention strategies to local needs, purchasing equipment

**Implementation**

1. How has the decentralization process unfolded in Zambia/ Province/District/facility/catchment area?
2. What some of the actors/ department involved the decentralizations process of the program province/ district? (Probe for NGOs, churches, traditional leaders etc)
   1. How were the different stakeholders engaged to be a part of the implementation?
   2. How do they work together to implement decentralized TB services?
   3. What are the strengths and weaknesses of the collaboration?
3. How are the coordination processes for the delivery of decentralized Drug-Resistant TB Services across partners / stakeholders done?
   1. What has worked well with this stakeholder coordination/ partnerships?
   2. What are gaps in the partnership/ coordination?
   3. How do issues of power and collective action affect the collaboration? Probe for trust, relationship building?
4. Are there any stakeholders that have been left? If yes, why? What can have done to bring them on board?
5. What can be done to improve the collaboration of stakeholders in implementing decentralization of Drug-Resistant TB Services?
6. How complex did you and your colleagues find the decentralized services to be?
   1. Do you feel as though you are in a good position to implement the services based on the strategies put in place before initial implementation?
7. What type of resources have been necessary for the implementation of decentralized MDR TB services? Probe for space, equipment, time, staff, program materials and source of resources.
8. Have you been implementing the decentralized services as they were intended?
   1. Why do you feel this is the case?
   2. What challenges are you facing that affect how well they implement the services as planned?
9. What are some of the policies which are relevant to and that guide or support implementation MDR TB (list them)?
   1. How do these policies affect the implementation of TB-MDR?
      - How do the policies support implementation of TB-MDR?
      - How do the policies hinder implementation of TB-MDR?
      - How can the policy environment be improved to better support the implementation of TB-MDR?
10. Have any changes been made to the decentralization policy and its implementation?
    1. What drove the need for these changes?
    2. How did the staff know which changes were the most appropriate?
11. What are some of the barriers and facilitators to the implementation of decentralized MDR TB services?

**Effectiveness of decentralization on MDR process, treatment and implementation outcomes**

1. Are you able to tell whether the decentralization is working? How can you tell?
   1. Has the decentralization process resulted in the recruitment of more patients?
   2. What are some of the positive consequences that you have observed
   3. What are some of the negative consequences that you have observed
2. What are some of the things that the patients have said since decentralisation?
3. How would you describe patient experiences in accessing MDR-TB care and treatment now that services have been decentralized?
   1. At the treatment centre? Probe for barriers and facilitators to accessing services.
   2. Within the community? Probe for barriers and facilitators to accessing services.
4. To what extent are patients adhering to treatment under the decentralized mode?
   1. Probe for barriers and facilitators to treatment adherence
5. Are the groups of patients for whom the decentralization has had less impact than other? Probe for specific categories and why that is?

**Success and challenges of decentralization**

1. What are some of the key successes and challenges to the implementation of decentralized MDR TB services in Zambia Province/district/facility/catchment area?

**Sustainment**

1. How has COVID-19 affected the implementation of decentralized MDR-TB services in Zambia/Province/district/facility? (To help assess the resilience of the program)
   1. Are there any contextual changes coming up in the foreseeable future that you think may have an impact on the delivery of decentralized services? Do you feel that you are able to deal with challenges in a better way since decentralization?
2. What is the likelihood that the implementation of the decentralized services is going to continue in the long term?
   1. Do you feel as though it will be integrated in to existing systems?
   2. What would be needed for the services to be delivered in the long term?

**Recommendations**

1. Based on your experiences, do you have any recommendations on how to improve the implementation of drug resistant TB services?

**Patient and Caregivers interview guide**

**Introduction**

1. Please tell me about yourself and your background?
2. Could you tell me a little about how you became diagnosed with drug resistant TB?
   1. Probe for when they started exhibiting symptoms, when they sought treatment, what prompted them to seek treatment?

**Decentralization and implementation processes**

1. Where did you receive diagnosis and treatment? Within the community or at a treatment centre?
   1. How did you end up going to the treatment centre?
   2. If at a treatment center, which treatment center did you attend? Probe for name and district. What services did you receive?
   3. For community, who provided the services and what services were given?
2. Were you required to go the treatment centre multiple times or did you receive some services within the community?

**Effectiveness of decentralization and determinants**

1. Was this your first time getting diagnosed with TB?
   1. If no, how did the treatment processes differ from the other times?
2. How would you describe your experiences in accessing MDR-TB care and treatment?
   1. At the treatment centre? Probe for barriers and facilitators to accessing services.
   2. Within the community? Probe for barriers and facilitators to accessing services.
   3. Who do you trust in delivering the services? Probe -providers, CHWs or nurse
   4. How does trust affect patients in accessing the services? How can trust and relationships between providers and clients be improved?
3. Have you completed your treatment for drug resistant TB?
   1. What aspects of the services you are receiving helped you adhere to the treatment the most?
   2. Probe for barriers and facilitators to treatment adherence
4. If you stopped without completing your treatment
   1. What changes to the services could have helped you adhere to your treatment regimen for longer?
   2. Probe for barriers and facilitators to treatment adherence
5. If you are currently undergoing treatment, how easy is it for you to adhere to the treatment?
   1. What aspects of the services you are receiving have helped you adhere to the treatment the most?
   2. Probe for barriers and facilitators to treatment adherence.
   3. Do you think you will continue with your regime?
6. Has receiving MDR-TB care and treatment had any impact on your life?
   1. Probe for positive and negative consequences?
7. Sometime back the MDR-TB services used to be provided in a health facility where a patient would be admitted until they completed their treatment. How do you think that would work for you?

a. Probe for advantages and disadvantages of decentralization

b. Probe for their preference between centralized and decentralized services. Ask why?

1. What do people in your community say about TB services? Are these things true or not? What is your opinion?

**Recommendations**

1. Based on your experiences, do you have any recommendations on how to improve the implementation of drug resistant TB services?
